# Comparison of the RAMSAY score and the Richmond Agitation Sedation Score for the measurement of sedation depth

**DOI:** 10.1186/cc10933

**Published:** 2012-03-20

**Authors:** R Riessen, R Pech, P Tränkle, G Blumenstock, M Haap

**Affiliations:** 1 University Hospital Tübingen, Germany

## Introduction

We implemented an interdisciplinary algorithm for the management of analgosedation in mechanically ventilated patients based on a new national guideline. As part of this project we investigated whether the newly introduced Richmond Agitation Sedation Score (RASS) allowed a better monitoring of sedation depth than the formerly used RAMSAY score.

## Methods

During the baseline phase of the study we investigated the RAMSAY score, which had been routinely used for several years in our unit. Following an educational program the RAMSAY was replaced by the RASS. During both study phases the actual sedation score was determined within a short period of time by the nurse in charge and an independent observer. In addition, the nurses were asked to evaluate on a six-point Likert scale whether the score appeared to be suitable to describe the actual state of sedation or to discriminate between different levels of sedation (1 = very good). The measurements took place at three defined time points (7, 9 and 12 o'clock) during the morning shift on weekdays.

## Results

In the baseline phase (36 patients/422 measurements) using the RAMSAY score, sedation depth documented by the nurses and the observer matched in only 39% of the measurements. The nurses documented in 246 (58%) measurements a lighter sedation and in 12 measurements (3%) a deeper sedation than the observer. In the post-implementation phase (37 patients/346 measurements) using the RASS, we found a significantly higher matching rate of 76% between nurses and observer compared to RAMSAY (*P *< 0.001). Nurses documented in 47 measurements a lighter (14%) and in 37 measurements (11%) a deeper sedation than the observer. The nurses evaluated the RASS in terms of the ability to describe the actual depths of sedation with a mean of 1.7 on the six-point Likert scale significantly better than the RAMSAY score with 3.2 (*P *< 0.001). Similar results were found regarding the discrimination between different levels of sedation (RASS 1.7, RAMSAY 3.1, *P *< 0.001).

## Conclusion

In routine use the RAMSAY score showed a poor performance regarding the measurement of sedation depth. After implementation of the RASS, measurement of sedation depth appeared significantly improved.

